# Immunoadsorption-Based HLA Desensitization in Patients Awaiting Deceased Donor Kidney Transplantation: An Interventional, Non-Randomised, Single Cohort Study

**DOI:** 10.3389/ti.2023.11212

**Published:** 2023-08-23

**Authors:** Côme Bureau, Cédric Rafat, Jean Luc Taupin, Stéphanie Malard, Laurent Mesnard, Hélène François, Camille Petit-Hoang, Nacera Ouali, Alexandre Hertig, Matthieu Jamme, David Buob, Eric Rondeau, Pierre Galichon, Yosu Luque

**Affiliations:** ^1^ Assistance Publique – Hôpitaux de Paris, Soins Intensifs Néphrologiques et Rein Aigu, Département de Néphrologie, Hôpital Tenon, Paris, France; ^2^ Assistance Publique-Hôpitaux de Paris, Laboratoire Régional d’Histocompatibilité, Hôpital Saint Louis, Paris, France; ^3^ Sorbonne Université, CoRaKid Inserm UMR_S1155, Paris, France; ^4^ Assistance Publique-Hôpitaux de Paris, Service d’Anatomie et Cytologie Pathologiques, Hôpital Tenon, Paris, France

**Keywords:** kidney transplant, graft survival, HLA desensitization, apheresis, immunoadsorption

## Abstract

Whether immunoadsorption (IADS) as part of desensitization protocols could facilitate deceased donor kidney transplantation (KT) in highly sensitized (HS) patients remains to be proven. We retrospectively analyzed our IADS based desensitization protocol for deceased donor KTs between 2013 and 2018. Fifteen HS patients (age 52 years [40–56]) were included. Waiting time before IADS was 6 years [5–10] and the interval between IADS initiation and KT was 5 months [1–12] for the 14 transplanted patients. Nine patients had prior KT. Calculated panel reactive antibody decreased significantly during the protocol (99.3% [92.5–99.9] vs. 79.4% [56.7–81.9]; *p* = 0.004). Death-censored graft survival was 85.7% at 1 and 2 years post-transplantation. One-year median plasma creatinine level was 135 µmol/L [111–202]. Six developed active antibody mediated rejection (ABMR) at 1 year, with a median delay of 13 days [11–26]. Eight patients developed severe infections, including two fatal outcomes. Finally, compared to 93% of patients who received desensitization receiving a KT, only 43% of a control with similar characteristics underwent transplantation. However, no difference was found in overall probability of being alive with a functioning graft at the end of follow-up. The results indicate that our IADS-based desensitization strategy was not effective due to a high rate of ABMR and severe infectious complications which pose a challenge to its universalization.

## Graphical Abstract

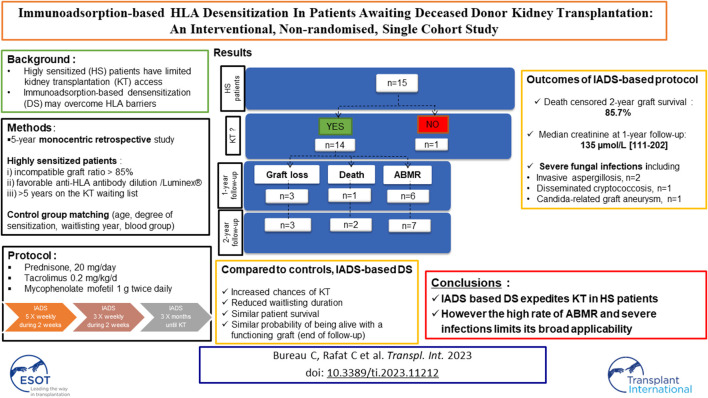

## Introduction

Kidney transplantation (KT) is universally acknowledged to be the treatment of choice for patients with end stage kidney disease (ESKD) in terms of survival and quality of life. In France, as elsewhere, the population affected by chronic kidney disease has grown steadily over the past decades totaling 51,000 patients with ESKD on dialysis and 9,675 on KT waiting lists in December 2021, whilst deceased donor organ procurement has plateaued, resulting in a shortage of organs [1].

Sensitization against HLA epitopes through blood transfusion, pregnancy and prior organ transplantation also hampers KT access resulting in protracted waiting time and increased mortality. Within the Eurogroup Transplantation Consortium it is thus estimated that 5% of patients are deemed highly sensitized (HS), as determined by an HLA antibody profile that reacts to ≥85%–100% of donors in the donor population [2]. The advent of novel assays providing enhanced HLA antibody detection—first and foremost being highly sensitive bead-based Luminex^®^ single antigen assays—has further increased the proportion of patients categorized as HS. In addition, the most recent studies have stressed the preeminence of donor specific antibodies (DSAs) as a predictor of post-KT active antibody mediated rejection (ABMR) and graft survival. They have prompted new risk stratification strategies and, in turn, novel therapeutic procedures designed to circumvent the negative outcomes occasioned by alloimmunization [3].

Desensitization protocols have emerged as one approach to overcome the HLA barrier and allow for KT in HS ESKD patients [4, 5]. Various strategies have been utilized but most protocols are built around pharmacological immunosuppression combined with apheresis. They also share a common goal, which is to deplete B cell populations and to reduce DSA to levels amenable to KT with a negative complement-dependent cytotoxic (CDC) crossmatch. Immunoadsorption (IADS), using Immunosorba^®^ columns (Globaffin^®^, Fresenius), has established itself as one of the preferred techniques among different apheresis options [6]. Compared to plasmapheresis, it provides semi-specific plasma treatment, superior immunoglobulin clearance, and obviates the need for albumin or plasma substitution [7]. IADS-based desensitization has shown good results for living donor KT [8]. There are few data in the setting of deceased donor KT where such an approach implies strict compliance with repeated sessions of apheresis and sustained immunosuppression pending allocation of an acceptable KT [9–11]. This study comprises a single center (Tenon Hospital, Paris, France) report of the outcomes associated with 15 consecutive HS patients who were on a kidney transplant waiting list and included in an IADS-based desensitization protocol.

## Methods

### Patient Population Selection and Definitions

We retrospectively analyzed all patients between January 2013 and September 2018, who underwent IADS-based HLA desensitization protocol for deceased donor KT. Patients deemed eligible for the procedure had to fulfill the following criteria: 1) an incompatible graft ratio >85% calculated for a given individual on the basis of his anti-HLA antibodies profile and the HLA pattern stemming from nationwide kidney procurement performed over the last 5 years, 2) a favorable anti-HLA antibody dilution test performed using the Luminex^®^ technique to mitigate a prozone effect and to predict adequate depletion through IADS, 3) more than 5 years on the KT waiting list, 4) protocol acceptance. Concerning antibody dilution test, before single antigen flow bead testing, a 0.1-M solution of disodium EDTA (Sigma-Aldrich^®^, St Louis, United States) at pH = 7.4 was diluted 1:10 in the sera and incubated for 10 min to avoid prozone effect. The French kidney allocation system offers organs at a national level. A national priority is given to highly sensitized patients based on their immunological profile (“incompatible graft ratio” >85%). During IADS protocol as the sensitization decrease that national priority can be removed. The French allocation system also allows for a locally retrieved kidney to be offered locally depending on match ability (ABO blood group and HLA compatibility measured by CDC crossmatch).

For each patient receiving the desensitization protocol, two control patients were selected from the same KT center. Controls were matched for age, degree of sensitization, had been waitlisted for a KT in the same year as the study patient, were of the same ABO blood group, and needed to have not died or be transplanted before the study patient had started the IADS protocol. Follow-up data for controls included KT status and date of KT and/or date of death.

Kidney biopsies were scored according to the 2017 Banff classification.

### Desensitization Protocol

The description of the desensitization protocol is presented in the Supplementary Methods S1.

### Immunoadsorption Therapy and Immunosuppressive Therapy for KT

The descriptions of the immunoadsorption therapy and the induction and maintenance immunosuppressive therapy for KT are presented respectively in the Supplementary Methods S2, S3.

### Anti HLA Antibodies and CDC Crossmatch Assessment

The description of the anti HLA antibodies and CDC crossmatch assessment is presented in the Supplementary Methods S4.

### Clinical Data

We obtained clinical data from medical records in our center and the CRISTAL database from the Agence de la Biomédecine. Each recipient from the present study gave written informed consent to be included in the CRISTAL database networks. The follow-up was terminated in August 2021.

### Statistical Analysis

Continuous variables were expressed as median (interquartile range) and categorical variables as numbers (percentages). Continuous variables were compared using the nonparametric two-tailed Mann-Whitney test. Qualitative variables were compared using the Chi squared test.

### Ethics Statement

The study was conducted in accordance with the ethical guidelines of the Assistance Publique—Hôpitaux de Paris. No institutional review board approval was necessary at the time of the study as it was a retrospective study involving no intervention. The study was conducted according to the ethical standards of the 2000 Declaration of Helsinki as well as the Declaration of Istanbul 2008.

## Results

### Demographics

From 2013 to 2018, a total of 15 HS ESKD patients were included in the IADS-based desensitization protocol for deceased donor transplantation in our center. During the same period, 497 kTs were performed in the center (66 from a living donor and 431 from a deceased donor). A total of (Table 1 and Supplementary Table S1) 4 men and 11 women with a median age of 52 years [40–56] were cleared for the protocol. Their median body mass index was 26 [21–29] and 12 out of 15 had African-Caribbean origins. Nine patients presented with hypertension and one with diabetes mellitus. All patients had a history of more than 3 blood transfusions. Women (*n* = 11) presented with a median 3 [2–5] previous pregnancies. The median duration of renal-replacement therapy was 11 years [8–14] and the median time on the waiting list was 6 years [5–10]. Nine patients had received either 1 (*n* = 6) or 2 (*n* = 3) previous KTs. Upon starting the desensitization program, patients exhibited a calculated panel reactive antibody (cPRA) of 99.3% [92.5–99.9] and an anti-class I PRA-CDC of 30% [18–41]. Kidney diseases are detailed in Supplementary Table S1. All were seronegative for HIV and HCV but six patients had a past HBV infection (positivity for anti-HBc and anti-Hbs antibodies) and two had a chronic HBV infection. In total, 12 out of 15 patients had received previous immunosuppressive therapy (for initial kidney disease or for previous KT). For all but one patient the dilution test performed on their serum showed a significant decrease in anti-HLA antibody titers.

**TABLE 1:** Demographic and nephrological features before transplantation.

Number of patients	*n* = 15
Demographic features	
Sex, male n (%)	4/15 (27)
Age, years	52 [40–56]
Ethnicity	
Sub Saharan African, *n* (%)	9/15 (60)
North African, *n* (%)	1/15 (7)
Caucasian, *n* (%)	3/15 (20)
Caribbean, *n* (%)	2/15 (13)
Sensitization-associated characteristics	
Previous kidney transplantation, *n* (%)	9 (60)
Number of pregnancies for women, *n* (%)	3 [2–5]
Transfusions > 3, *n* (%)	15/15 (100)
cPRA, %	98 [88–99]
PRA-CDC (anti-HLA class I), %	30 [18–41]
Historical positive CDC Crossmatch	8/15 (53)
Nephrological features pre-Tx	
Initial kidney disease, n (%)	
Undetermined	3 (20)
Nephrosclerosis	3 (20)
Membranous nephropathy	1 (6.7)
Anti-GBM disease	1 (6.7)
ADPKD	1 (6.7)
FSGS	1 (6.7)
Chronic hemodialysis duration, years	11 [8–14]
Time span between waiting list registration and IA initiation, years	6 [5–10]
Donor’s characteristic	
Age, years	65 [37–70]
Hypertension, *n* (%)	3/14 (21)
Diabetic, *n* (%)	2/14 (14)
Serum creatinine, µmol/L	69 [56–84]
Proteinuria, g/24 h	0 [0–0.16]

Abbreviations: ADKP, autosomal dominant polycystic disease; CDC, complement-dependent cytotoxic; cPRA, calculated panel reactive antibodies; FSGS, focal segmental glomerulosclerosis; GBM, glomerular basal membrane; IA, immunoadsorption; Tx, transplantation.

ID: immunoadsorption-based desensitization waiting time: time elapsed between transplantation list registration and transplantation; waiting time after IA initiation: time elapsed between immunoadsorption-based desensitization and transplantation.

### Impact of Desensitization Protocol

**FIGURE 1:**
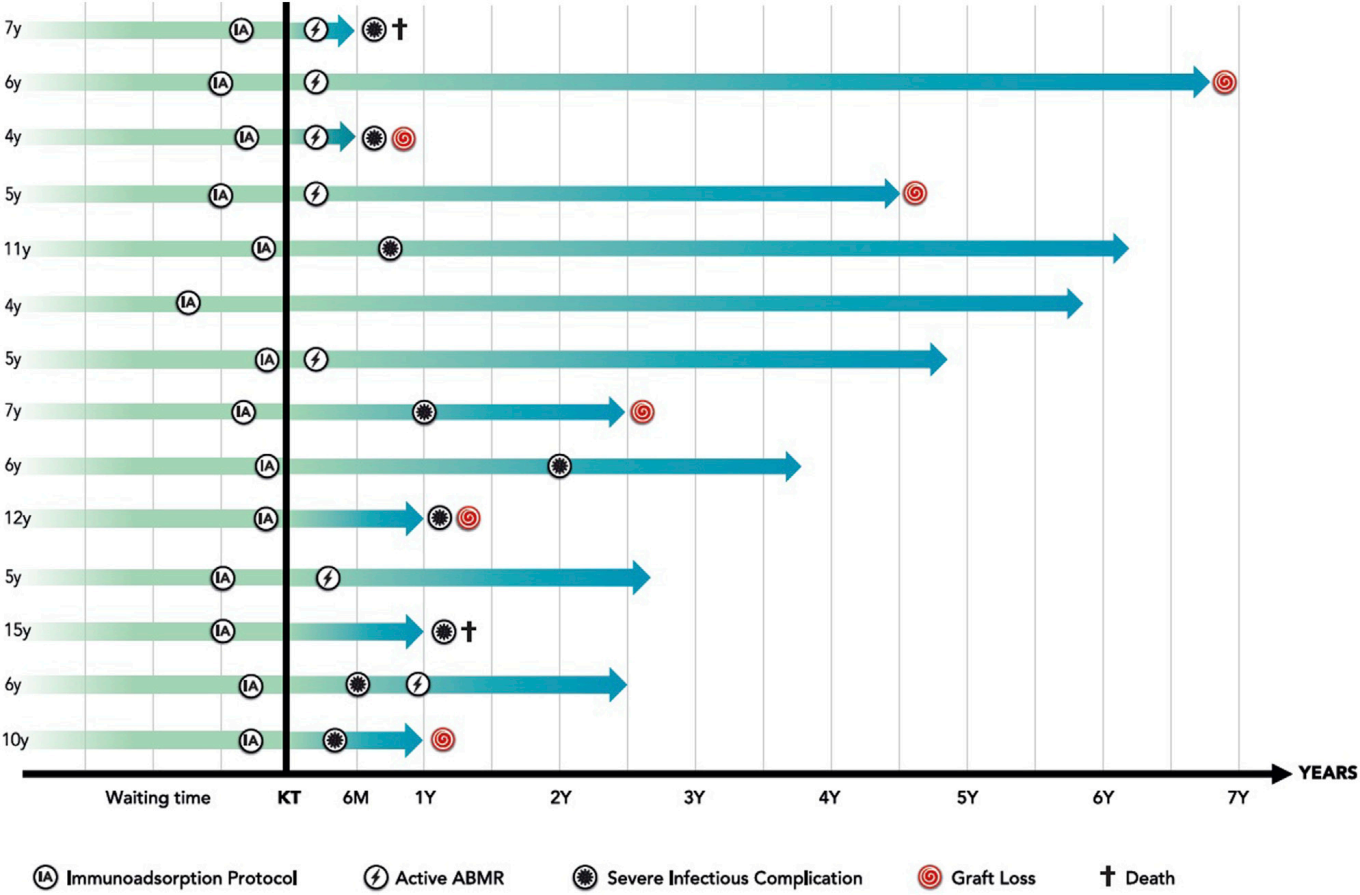
Immunoadsorption based desensitization protocol: overall results.

After initiation of the IADS-based desensitization protocol, KT was performed after a median of 5 months [1–12] in 14 out of 15 patients (Figure 1). The patients received a median of 23 IADS sessions [14–32] over a median of 110 days [35–141] before KT. All the patients received IADS using an arteriovenous fistula. cPRA fell significantly from 99.3% [92.5–99.9] before IADS to 79.4% [56.7–81.9] following completion of the final IADS session (*p* = 0.004). Side effects observed during desensitization were mycophenolate-induced diarrhea (*n* = 4), hypocalcemia (*n* = 3) and cytopenias (*n* = 1). In one case the IADS-based protocol was terminated after 19 sessions (2 months) due to diarrhea and cytopenia but the response in terms of anti-HLA antibody titer was favorable and the patient was transplanted 8 months later. The protocol was discontinued in one case after 16 sessions (1 month) due to a lack of efficacy—the cPRA remained at 100%. For all desensitized patients who were transplanted, the day 0 CDC crossmatch was negative. However, 7 out of 14 KT recipients had displayed a historic CDC positive crossmatch (3/14 IgG against T and B lymphocytes, 4/14 IgM only). Flow cytometry crossmatch is not routinely performed in France for deceased donor transplantation. The median cumulated historical DSA MFI value before transplantation was 21,222 [12,067–42,095] in class I and 6,157 [1,730–20,455] in class II antibodies. At the day of transplantation, median DSA number and sum total MFI of DSA were 3 [1.8–4.3] and 7,625 [2,771–10,201], respectively.

### Donor Characteristics

The median donor age was 66 years [40–71], and a history of hypertension and diabetes mellitus was disclosed in 3/14 (21%) and 2/14 (14%) patients, respectively. Donor serum creatinine was 69 μmol/L [56–84]. A cerebrovascular event was the recorded cause of death in 10 of 14 (71%) donors. The median number of HLA mismatches was 5 [5–6]. All KT recipients displayed at least one DSA on the day of transplantation: cumulative day 0 DSA-MFI was 4,505 [2,133–7,125] for class I and 1,150 [0–4,320] for class II. Median cold ischemia time was 15.5 h [12.5–18.5].

### Transplant Follow-Up

The median post-transplant follow-up was 3.1 years [1.7–4.9]. At 3 months post-transplant, cumulative DSA-MFI was 5,962 [4,229–11,200] for class I and 5,209 [2,599–8,593] for class II antibodies. Individual post-transplant DSA kinetics is shown in Supplementary Figure S1. Active ABMR was diagnosed in 7/14 (50%) patients (six within the first-year post-transplant). For two patients, active ABMR was subclinical and diagnosed based on protocol biopsies. All 7 patients were treated with steroids, plasma exchange or IADS, combined with eculizumab (*n* = 3), and/or IVIg (*n* = 6). Histopathological Banff scores are shown in Table 2. In 3/14 cases chronic ABMR was diagnosed during follow-up. Banff score on the available 3rd month protocol biopsies is shown in Supplementary Table S2.

**TABLE 2:** Post-kidney transplantation active ABMR episodes.

Patient	Delay between KT and active ABMR diagnosis (days)	Banff classification	Plasma creatinine at biopsy (µmol/L)	Urine protein to creatinine ratio (g/mmol)	Treatment	Outcome	Biopsy indication
1	8	g3 i0 t0 v0 ptc2 cg0 mm1 ci0 ct0 cv1 ah0 C4d3	282	NA	IADS, PE, steroids, IVIg	Sepsis and bleeding Death < M3	AKI
2	10	g2 i1 t0 v0 ptc1 cg0 mm0 ci0 ct0 cv1 ah0 C4d3	376	NA	IADS, eculizumab, steroids, IVIg	Chronic ABMR	AKI
4	12	g2 i2 t1 v0 ptc2 cg0 mm0 ci0 ct0 cv0 ah0 C4d0	697	0.17	PE, steroids, eculizumab, IVIg	Graft loss < M3	AKI
5	30	g2 i0 t0 v0 ptc1 cg0 mm0 ci0 ct0 cv2 ah0 C4d3	183	0.02	Steroids, PE, IVIg	Chronic ABMR Graft loss Y4	AKI
7	13	g2 i1 t0 v1 ptc2 cg0 mm0 ci0 ct0 cv0 ah0 C4d3	170	0.05	PE, eculizumab, steroids, IVIg		AKI
11	95	g1 i0 t0 v0 ptc0 cg0 mm0 ci0 ct0 cv1 ah0 C4d3	200	0.01	PE, steroids, IVIg		Protocol month 3
13	398	g2 i0 t0 v0 ptc0 cg0 mm1 ci0 ct0 cv0 ah0 C4d0	116	0.01	PE, steroids		Protocol month 12

Abbreviations: ABMR, antibody mediated rejection; AKI, acute kidney injury; IADS, immunoadsorption; IVIg, intravenous immunoglobulins; KT, kidney transplantation; NA, not available; PE, plasma exchange; g, glomerulitis; i, interstitial inflammation; t, tubulitis; v, intimal arteritis; cpt, peritubular capillaritis; cg, transplant glomerulopathy; mm, mesangial matrix increase; ci, interstitial fibrosis; ct, tubular atrophy; cv, arterial fibrous intimal thickening; ah, hyaline arteriolar thickening.

At 1-year post-KT, 2 KT recipients had died from severe infections but with functioning grafts and there were two graft losses (one due to recurrent focal and segmental glomerulosclerosis [FSGS] and one due to active ABMR). For the 10 functional grafts at 1-year, median serum creatinine and estimated glomerular filtration rate (eGFR) were 135 μmol/L [111–202] and 47 mL/min/1.73 m [3] [29–51], respectively. Uncensored graft survival was 71.4% at both 1 and 2 years. Death-censored graft survival was 85.7% at the same time points. By the end of study follow-up, 8/14 patients had lost their graft due to chronic allograft dysfunction (*n* = 3), death (*n* = 2), acute rejection (*n* = 1), renal arterial mycotic aneurism (*n* = 1), and FSGS recurrence (*n* = 1). There were a number of infectious complications that are listed in Supplementary Table S3.

### Comparing Outcomes of KT-Waitlisted Highly-Sensitized ESKD Patients With or Without IADS Desensitization

Finally, we compared outcomes between our 15 HS ESKD patients receiving IADS desensitization and a group of patients matched for age, degree of HLA sensitization and time of KT waitlisting (*n* = 30: 2:1) (Table 3). Compared to 93% of patients who received desensitization receiving a KT, only 43% of our control group underwent transplantation. Time from waiting list enrollment to KT was 6.5 [5.7–10.2] years in desensitized patients and 10.5 [8.3–11.7] years in controls. However, we did not find a significant difference in overall patient survival (87% vs. 96%, *p* = ns) and in the percentage of patients alive with a functioning graft (40% in both groups) at the end of follow-up.

**TABLE 3:** Comparing outcomes of KT-waitlisted highly-sensitized ESKD patients with or without IADS desensitization.

	IADS group	Controls	*p*
	*n* = 15	*n* = 30	
Demographics			
Age, years	52 [40–56]	52 [43–58]	0.68
ABO group, *n* (%)	AB 3/15 (20%)	AB 4/30 (13%)	0.67
	O 7/15 (47%)	O 19/30 (63%)	0.35
	A 3/15 (20%)	A 4/30 (13%)	0.67
	B 2/15 (13%)	B 3/30 (10%)	0.98
Degree of sensitization (TGI, %)	99 [92–100]	98 [72–99]	0.11
Outcomes at the end of follow-up			
Transplantation, *n* (%)	14/15 (93%)	13/30 (43%)	0.001
Time from waitlisting to KT, years	6.5 [5.7–10.2]	10.5 [8.3–11.7]	0.07
Death-censored graft loss, *n* (%)	6/15 (40%)	1/30 (3%)	0.003
Death, *n* (%)	2/15 (13%)	2/30 (6%)	0.85
Alive and functioning graft, n (%)	6/15 (40%)	12/30 (40%)	ns

Abbreviations: IADS, immunoadsorption; TGI, “taux de greffons incompatibles,” French sensitization score « percentage of incompatible kidney transplants »; KT, kidney transplantation.

The results are summarized in Figure 1.

## Discussion

Management of highly sensitized ESKD patients represent a conundrum for KT teams. It is well recognized that patients who are denied a KT have an increased mortality compared to recipients of an HLA incompatible KT, and this holds true in the presence of a historic positive cytotoxic crossmatch [12]. In addition, in the current kidney allocation system, anti-HLA sensitization decreases the chances of patients being allocated a kidney graft and may even preclude KT as in the case of some highly sensitized patients [13]. Conversely preformed DSA are acknowledged to expose patients to an increased risk of graft failure and ABMR [14].

Several strategies aimed at reducing anti-HLA antibody levels and enhancing the chances of HS patients being offered a KT have been elaborated. Early protocols utilizing exclusively IVIg [4, 15] have been replaced by those combining apheresis—either plasma exchanges [16–18] or IADS [6, 9, 10, 19, 20]—with immunosuppressive drugs (steroids, calcineurin inhibitors, mycophenolate mofetil, eculizumab, rituximab), and IVIg [21]. Recently, IdeS an IgG degrading endopeptidase has been shown to allow for greater anti-HLA antibody depletion after a single dose thus representing another potential option for HS transplant candidates in the near future [22, 23]. Imlifidase dispenses with the repeated and cumbersome IADS sessions and allows for a greater reduction in DSA, at least on the day of KT. Besides, it is effective in even the most highly sensitized patients and 3-year follow-up graft survival was encouraging (90%) [24].

With regards to apheresis techniques, IADS has been shown to be more efficient than plasma exchange for lowering anti-HLA antibody titers [22, 25], and obviates the need for plasma replacement with its attendant side effects. To date, the implementation of IADS-based protocols has been chiefly restricted to living donor HLA and ABO incompatible KT. Our data is a further contribution to the few prior experiences in the setting of deceased donors [8–10]. Our protocol led to a decrease in cPRA so that 93% of the patients were ultimately transplanted. These patients had been on the waiting list for several years with a low likelihood of ever receiving a KT. Our comparison with a relevant control group suggests that the desensitization protocol used here increases the probability of HS patients being transplanted and also expedites KT.

Only few experiences with IADS-based desensitization have been reported so far. Using a protocol akin to ours, Noble et al reported on 36 patients including 8 living donors. In six cases (16.7%) the IADS protocol was aborted due to failure to clear DSA or complications. With a different approach [9, 10] patients displaying a positive complement-dependent cytotoxicity crossmatch received a single session of IADS immediately prior to KT and were cleared for transplantation provided the crossmatch was rendered negative. Compared to our cohort, the patients exhibited lower HLA sensitization, and a significant proportion of the patients were deemed unsuitable for KT having failed to yield a negative crossmatch (around 20%). However, the study disclosed favorable graft survival rates.

However, the shortcomings of desensitization protocols should be recognized. While these have been instrumental in offering a therapeutic opportunity for HS patients, post-KT DSA rebound significantly increases the risk of ABMR as shown in the Supplementary Figure S1. Fifty percent of patients suffered active ABMR, and there were three cases of chronic ABMR. One team [26] (Schwaiger et al.) opted for systematic post KT IADS, yet the group of highly sensitized patients CDCXM+/DSA+ patients which most closely resembles our cohort nonetheless exhibited increased rates of ABMR (44%). In fact, irrespective of the desensitization approach, the rate of ABMR was a cause of concern ranging from 38% [24] to 41% [11]. From an immunological perspective, half of our patients were free of adverse immunological events post-KT despite high DSA levels. Taken together, these results suggest that 1) for any given patient, DSA alone should not preclude KT; 2) within the group of HS patients, current immunological risk stratifiers may incorrectly classify these patients as untransplantable. 3) the same stratifiers are ineffective at delineating HS patients who may enjoy a satisfactory post-KT course from those at risk of early ABMR.

We observed significant infectious complications in our cohort. This is unsurprising for a number of reasons. HS ESKD patients may have impaired immunity due to previous immunosuppression for native kidney disease, previous KT and on top of the burden of dialysis and ESKD itself. The multi-targeted immune desensitization protocol used here would have further enhanced the infectious risk. The high rate of invasive fungal infections (*n* = 4) including two fatal cases of aspergillosis and one case of disseminated cryptococcosis is a likely reflection of the patients’ defective adaptive cellular immunity [27–29]. In line with this, infection has been highlighted as the principal contributor to death in other cohorts of IADS-desensitized patients [26].

We recognize the limitations of the current study. Our data are observational and from a single center with a modest number of patients. However, these types of patients, that is those who are HS and who have been waitlisted for a significant amount of time, are uncommon. Nevertheless, as the fraction of HS patients is expected to grow over the coming years there is a dire need to devise strategies to raise the prospects of KT. Currently, there is no consensus on how to manage this very high-risk group resulting in divergent strategies around the world. Unfortunately, outcomes for these patients are dismal and so transplantation remains their only hope, albeit with the risks described here.

When contemplating IADS-based desensitization KT the risks entailed by remaining on the waiting list should be carefully weighed against the hazards of a potentially short-lived graft function, the high likelihood of ABMR and severe infections. Importantly, immunoadsorption strategy is not associated with an improved probability of being alive with a functioning graft at the end of the follow-up compared to those receiving no immunoadsorption. Indeed, those who received immunoadsorption had poorer graft outcomes following transplantation.

In our experience, the exceedingly high risk of ABMR and lethal infections outweighed the potential benefits of KT, precluding the universalization of our IADS-based desensitization strategy in its current scheme. However, there may be select groups of patients that might benefit from immunoadsorption and these should be defined in future studies. Single IADS, or better yet imlifidase, may represent less cumbersome options. Regardless of the adopted strategy, clinicians should be wary of the high rate of ABMR and candidates should be selected and informed accordingly.

## Data Availability

The raw data supporting the conclusion of this article will be made available by the authors, without undue reservation.
