# Supplementary material for: Immunoadsorption-Based HLA Desensitization in Patients Awaiting Deceased Donor Kidney Transplantation: An Interventional, Non-Randomised, Single Cohort Study
Source: Transpl Int. 2023 Aug 23;36:11212. doi: 10.3389/ti.2023.11212 (PMC10481532; doi:10.3389/ti.2023.11212)
Supplement: Supplementary file 1 [file DataSheet2.pdf]

Current transplantation DSA

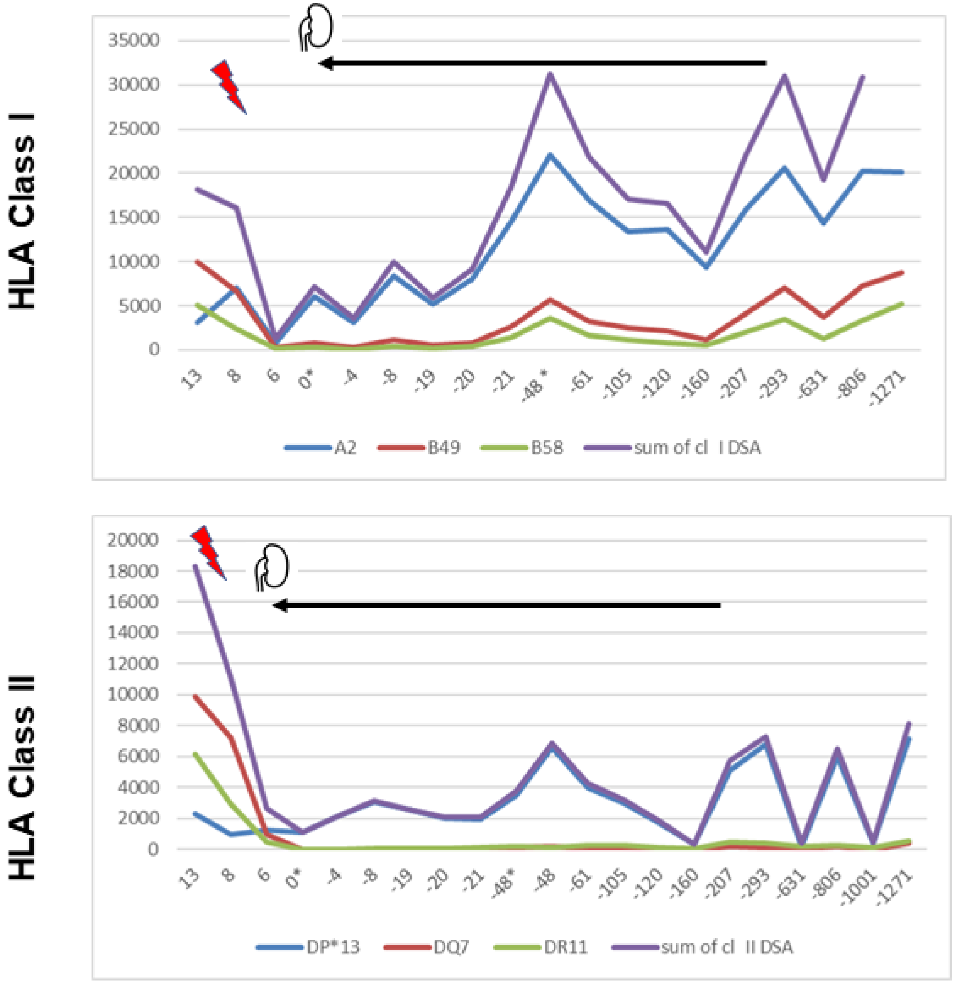

Patient 1

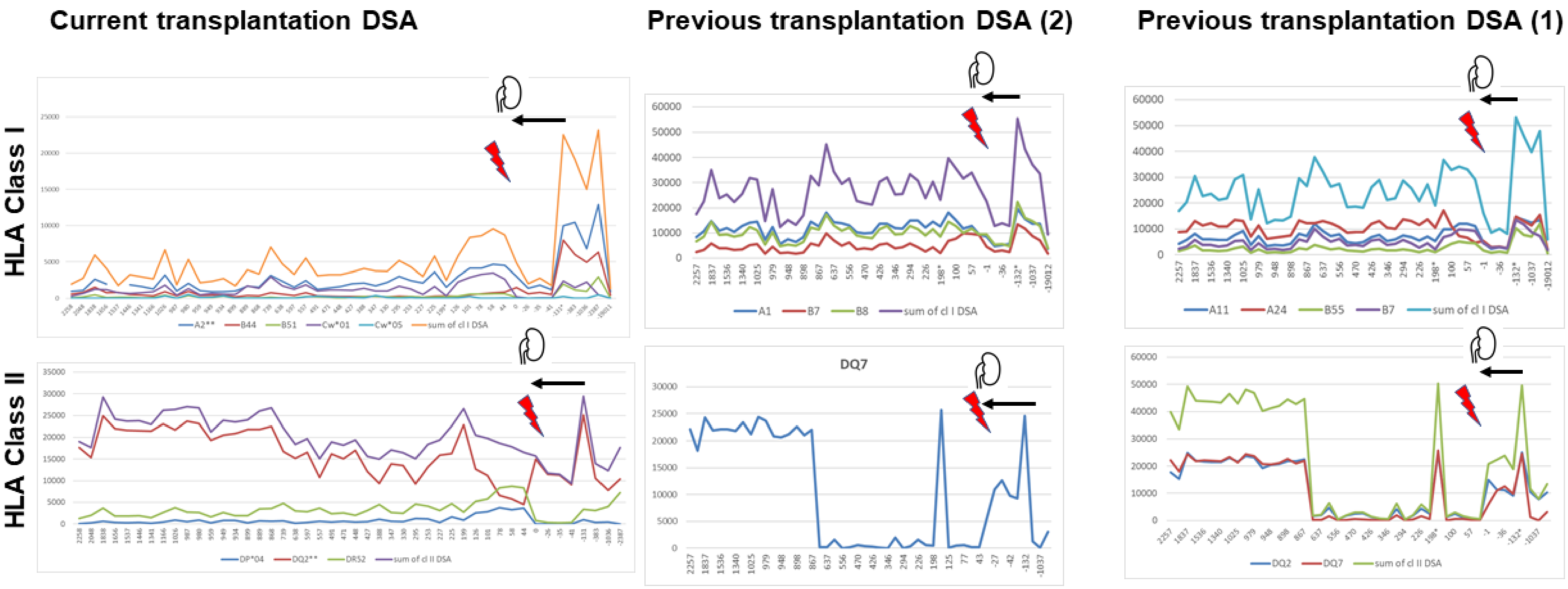
Current transplantation DSA
Previous transplantation DSA (2)
Previous transplantation DSA (1)
HLA Class I
HLA Class II
A2** B44 B51 Cw*01 Cw*05 sum of cl I DSA
DP*04 DQ2** DR52 sum of cl II DSA
A1 B7 B8 sum of cl I DSA
DQ7
A11 A24 B55 B7 sum of cl I DSA
DQ2 DQ7 sum of cl II DSA

Patient 2

Current transplantation DSA

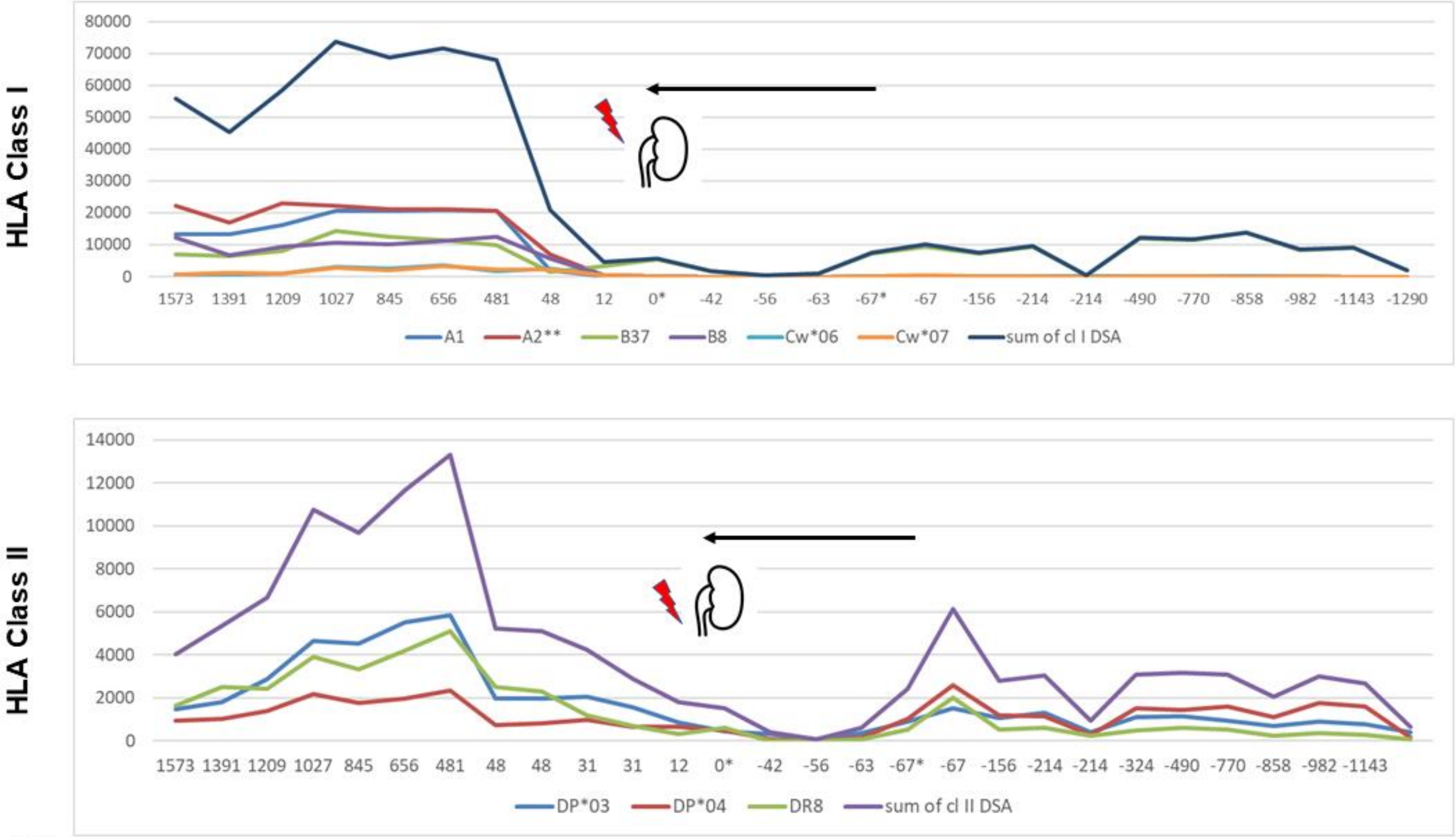

Patient 3

Current transplantation DSA

HLA Class I

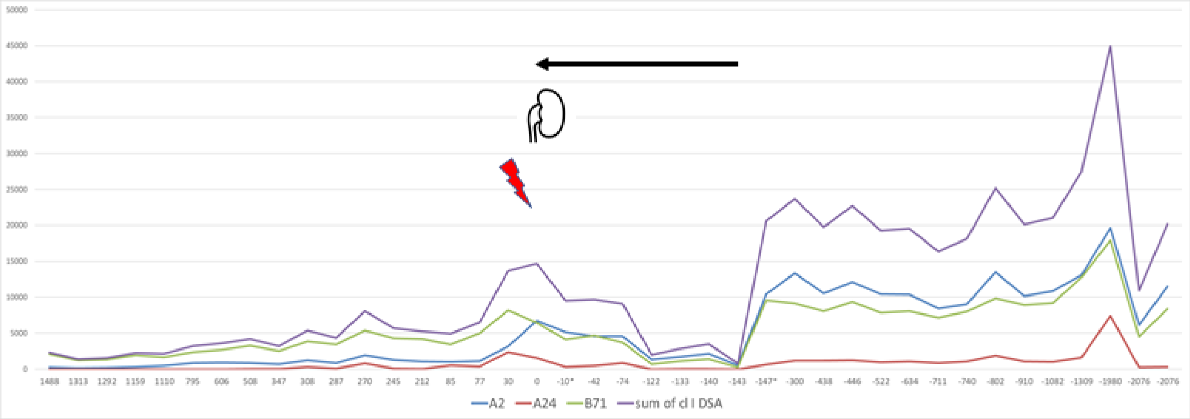

HLA Class II

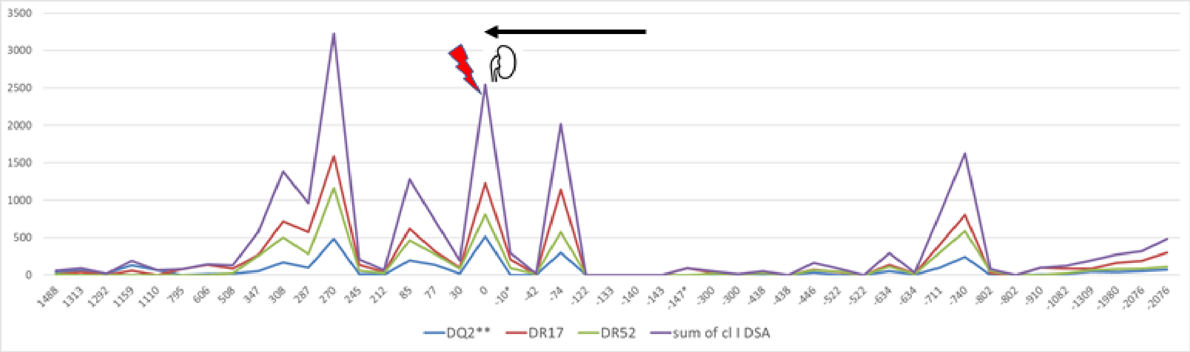

Patient 4

Current transplantation DSA

HLA Class I

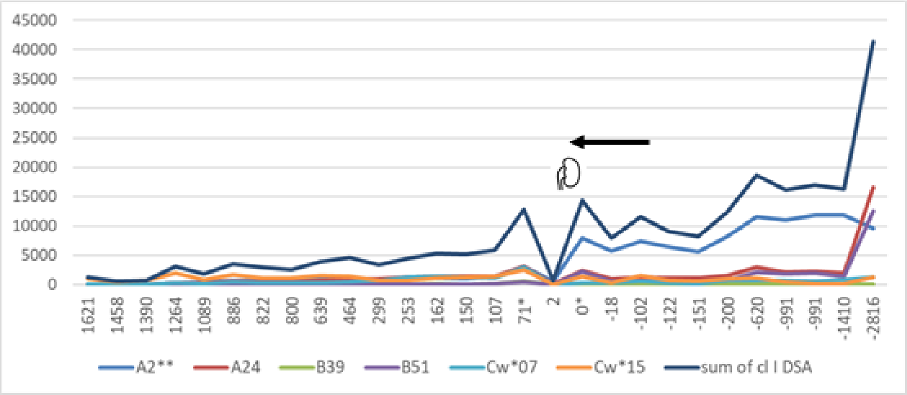

HLA Class II

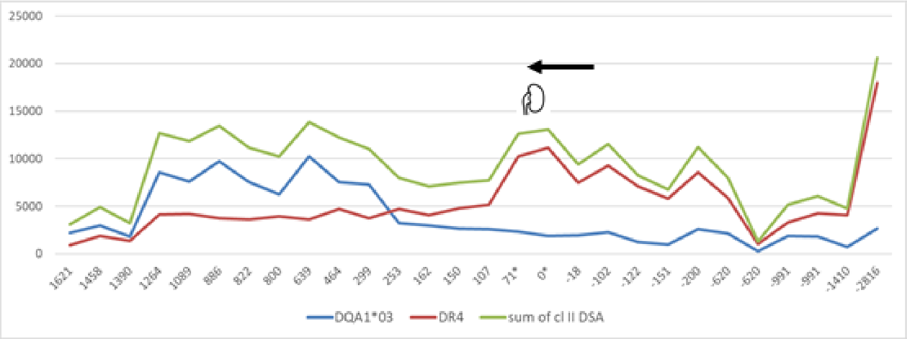

Patient 5

Current transplantation DSA

Previous transplantation DSA

HLA Class I

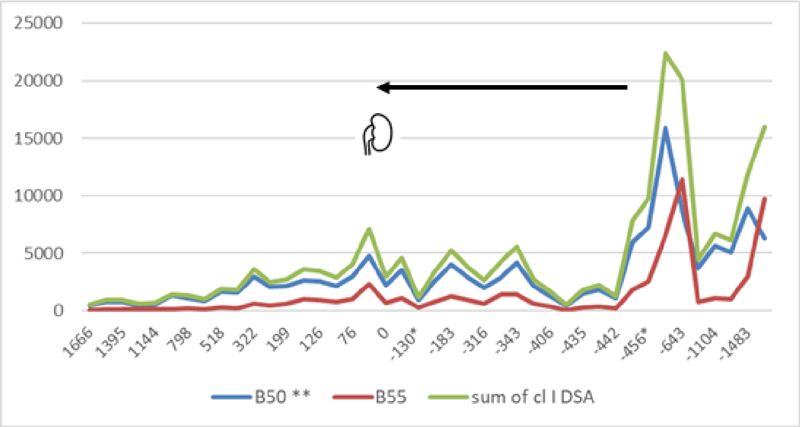

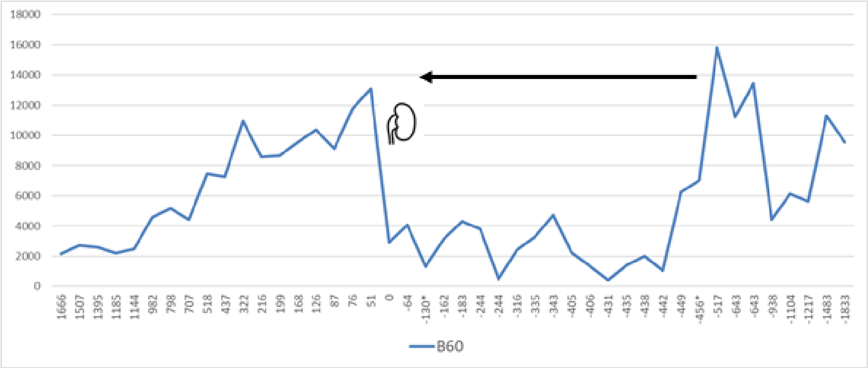

HLA Class II

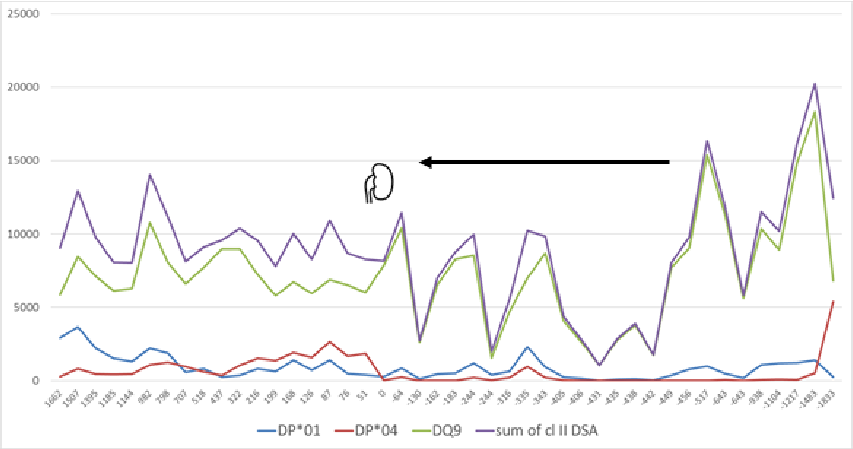

Patient 6

Current transplantation DSA

Previous transplantation DSA

HLA Class I

HLA Class II

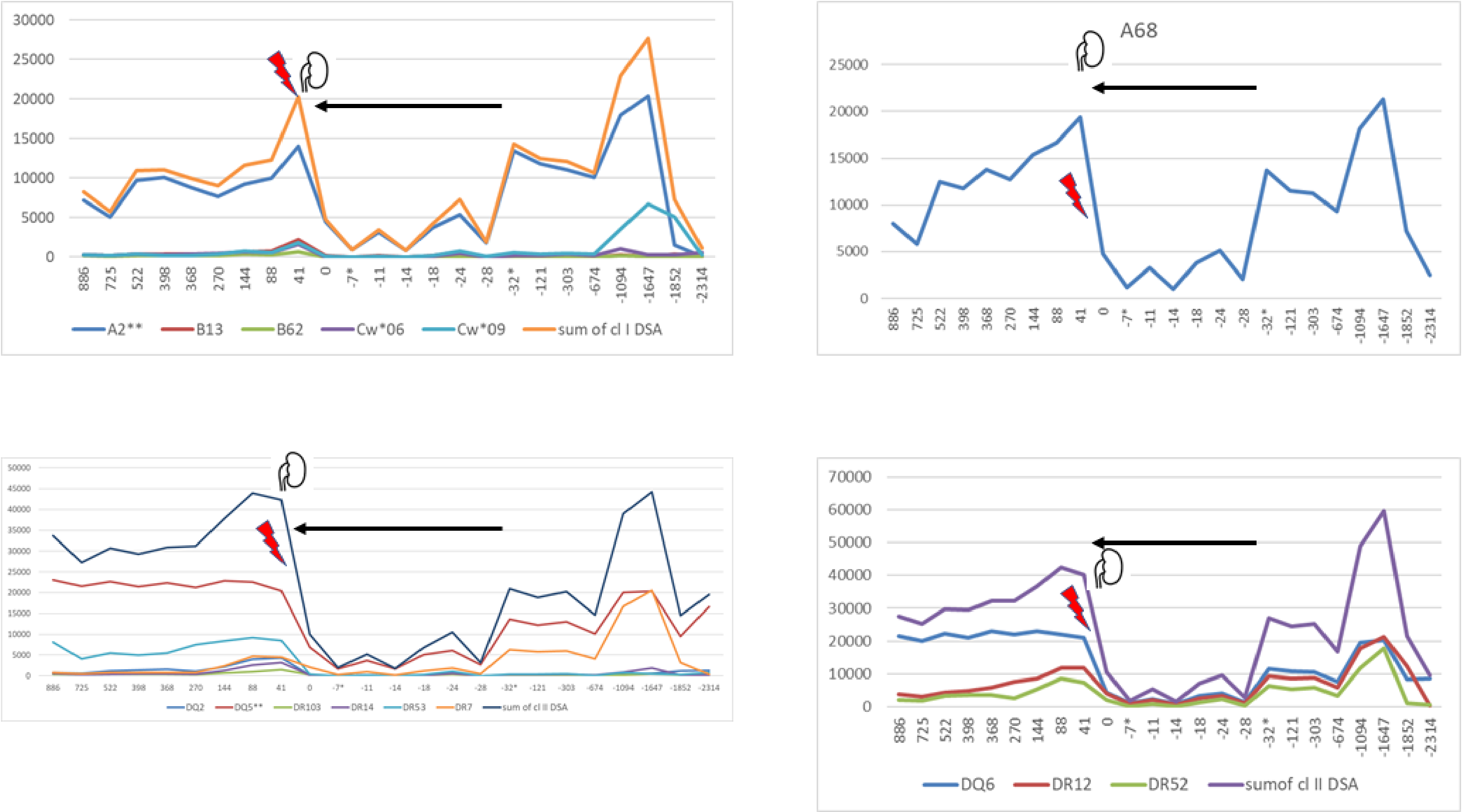

Patient 7

Current transplantation DSA

Previous transplantation DSA

HLA Class I

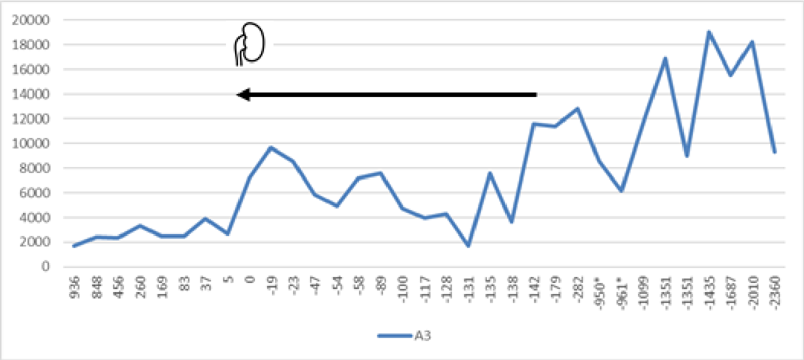

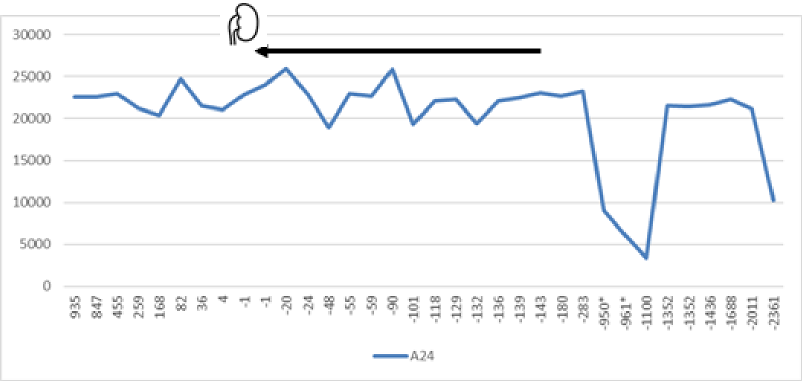

HLA Class II

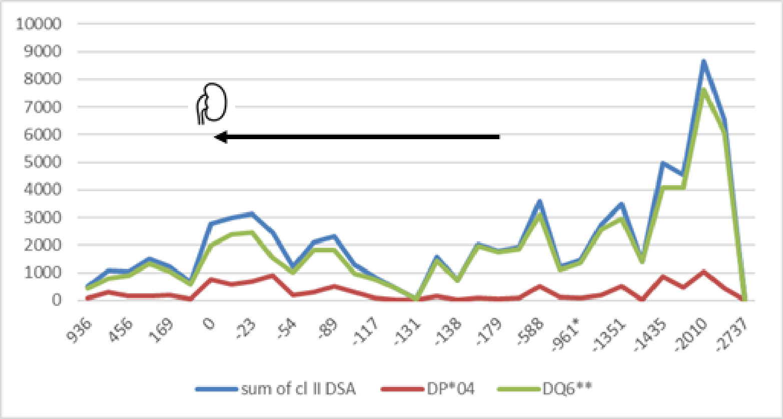

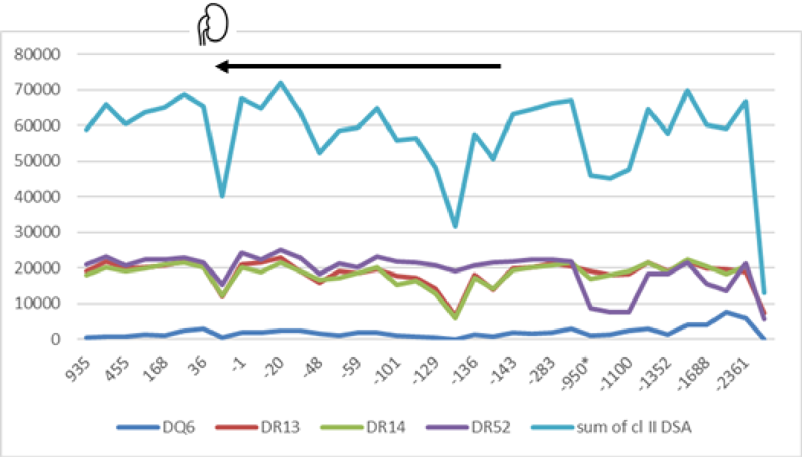

Patient 8

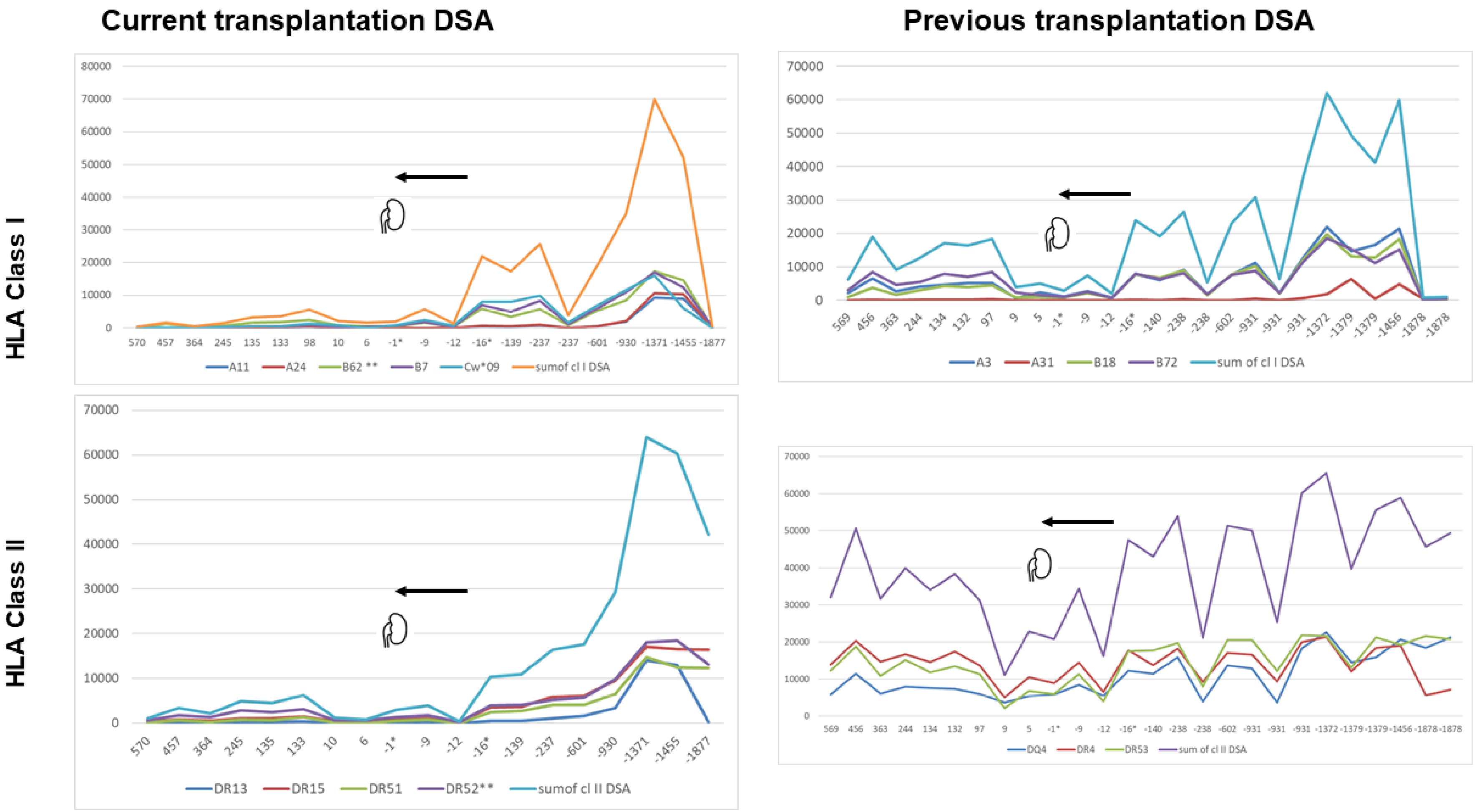
Current transplantation DSA
Previous transplantation DSA
HLA Class I
HLA Class II
570 457 364 245 135 133 98 10 6 -1* -9 -12 -16* -139 -237 -237 -601 -930 -1371 -1455 -1877
A11 A24 B62 ** B7 Cw*09 sumof cl I DSA
569 456 363 244 134 132 97 9 5 -1* -9 -12 -16* -140 -238 -238 -602 -931 -931 -931 -1372 -1379 -1379 -1456 -1878 -1878
A3 A31 B18 B72 sum of cl I DSA
570 457 364 245 135 133 10 6 -1* -9 -12 -16* -139 -237 -601 -930 -1371 -1455 -1877
DR13 DR15 DR51 DR52** sumof cl II DSA
DQ4 DR4 DR53 sum of cl II DSA

Patient 9

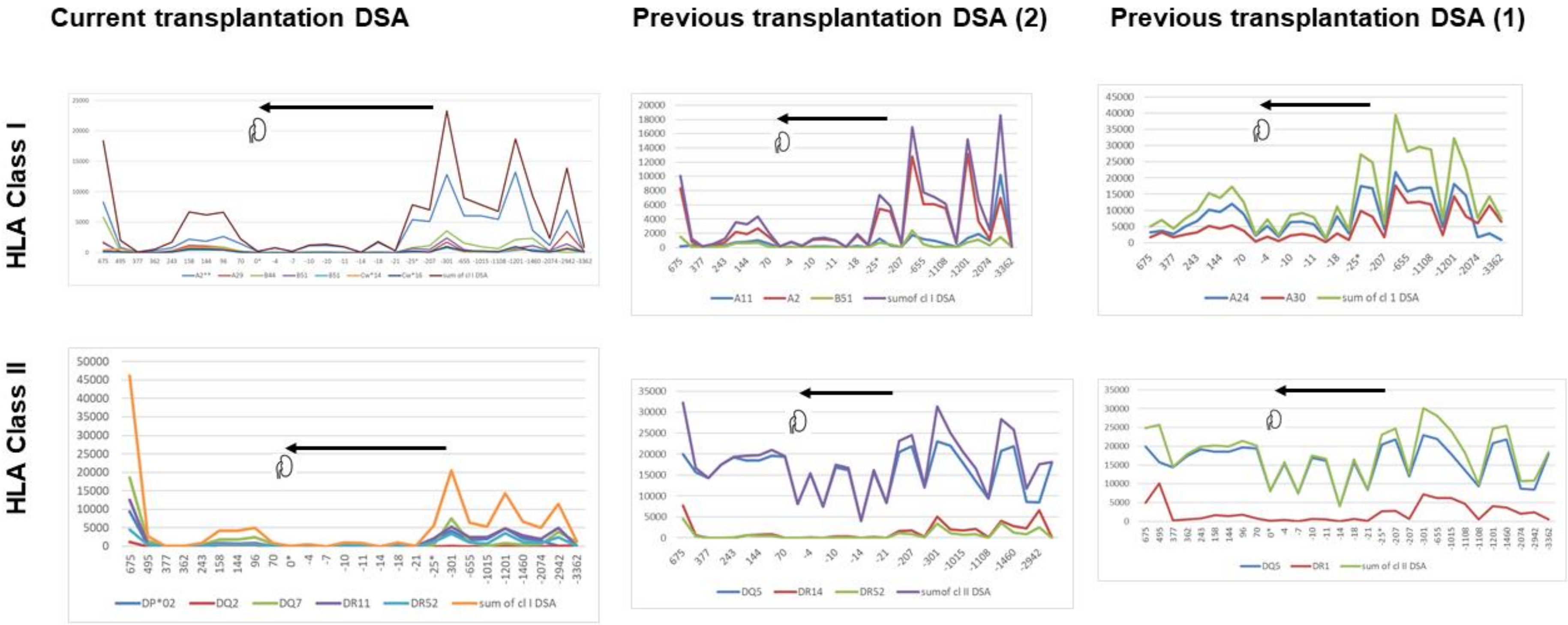
Current transplantation DSA
Previous transplantation DSA (2)
Previous transplantation DSA (1)
HLA Class I
HLA Class II
A11 A2 B51 sumof cl I DSA
A24 A30 sum of cl 1 DSA
DP*02 DQ2 DQ7 DR11 DR52 sum of cl I DSA
DQ5 DR14 DR52 sumof cl II DSA
DQ5 DR1 sum of cl II DSA

Patient 10

Current transplantation DSA | **Previous transplantation DSA**

HLA Class I

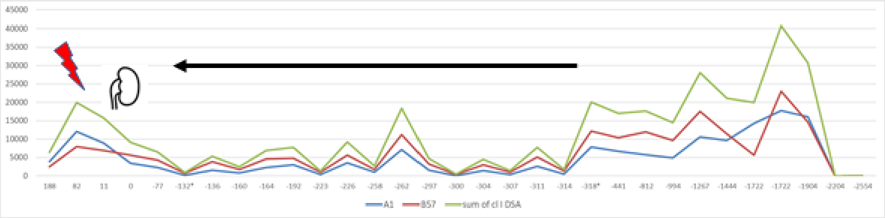

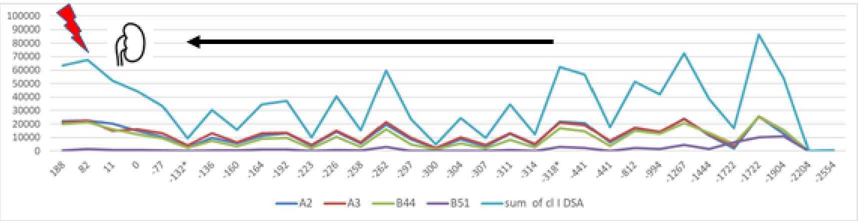

HLA Class II

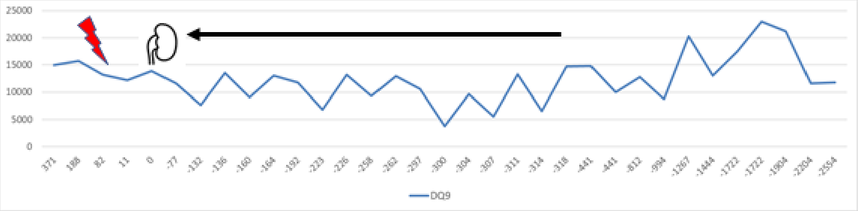

Patient 11

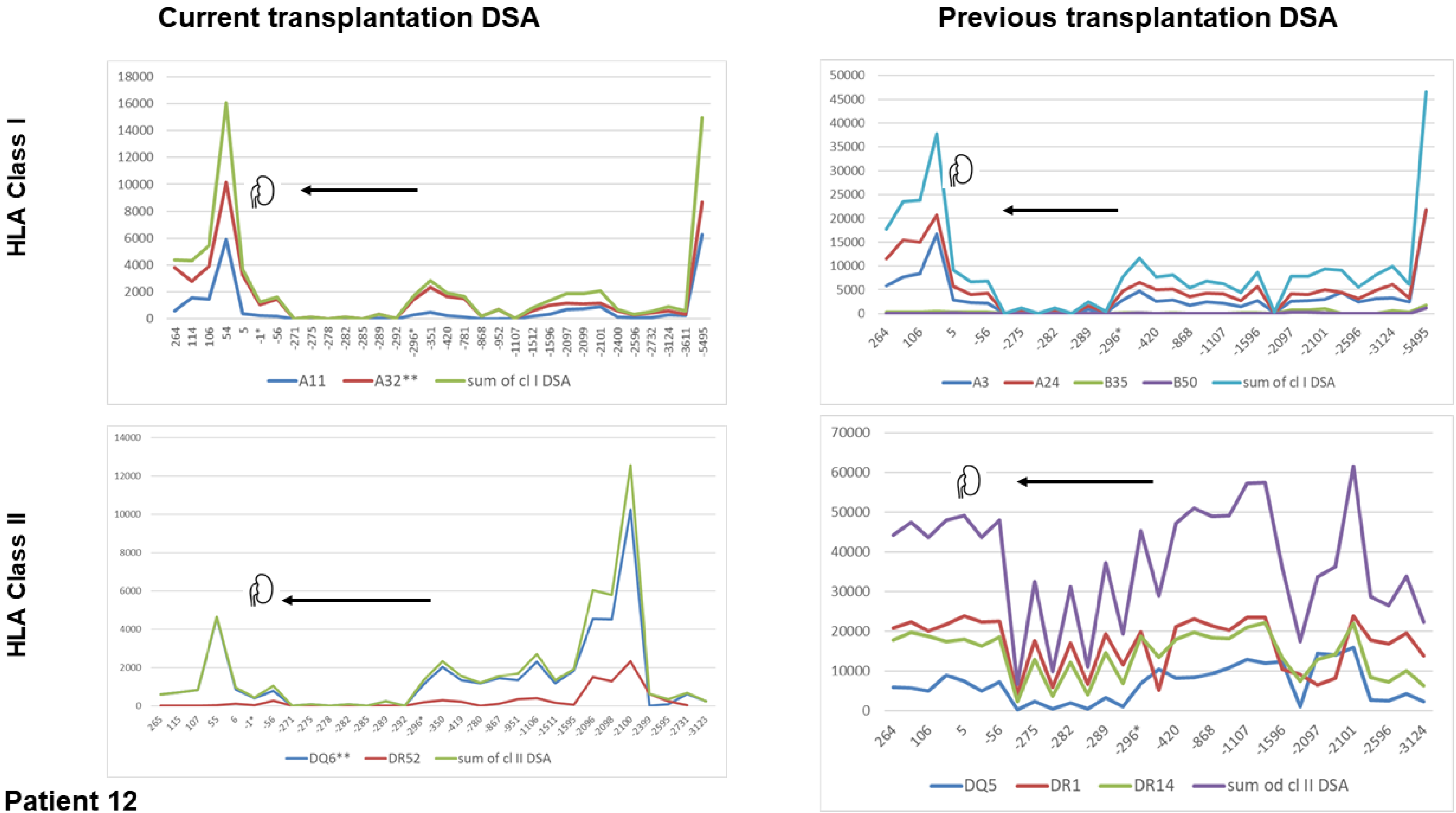
Current transplantation DSA
Previous transplantation DSA
HLA Class I
HLA Class II
A11
A32**
sum of cl I DSA
DQ6**
DR52
sum of cl II DSA
A3
A24
B35
B50
sum of cl I DSA
DQ5
DR1
DR14
sum od cl II DSA

Patient 12

Current transplantation DSA

HLA Class I

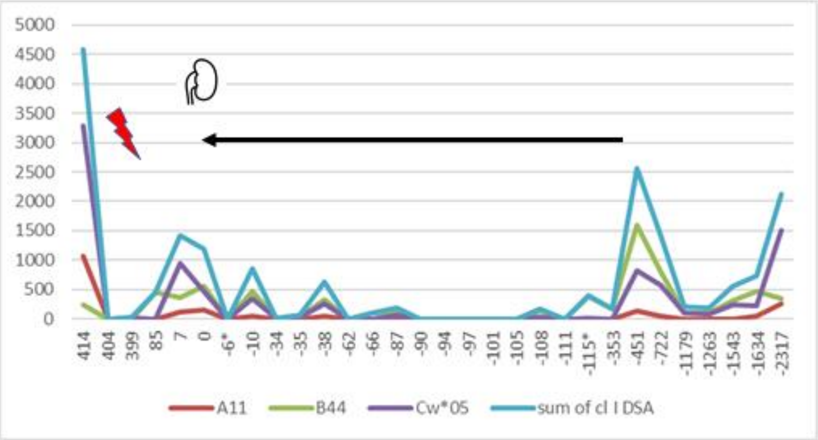

HLA Class II

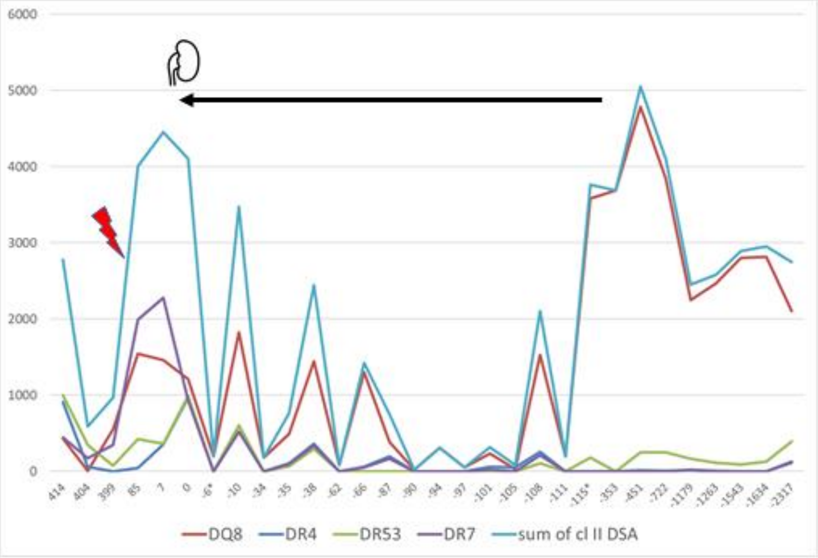

Patient 13

Current transplantation DSA

HLA Class I

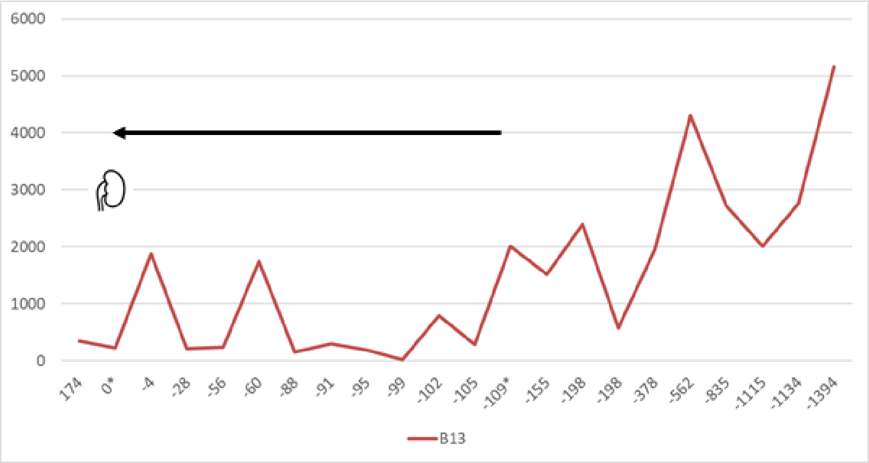

HLA Class II

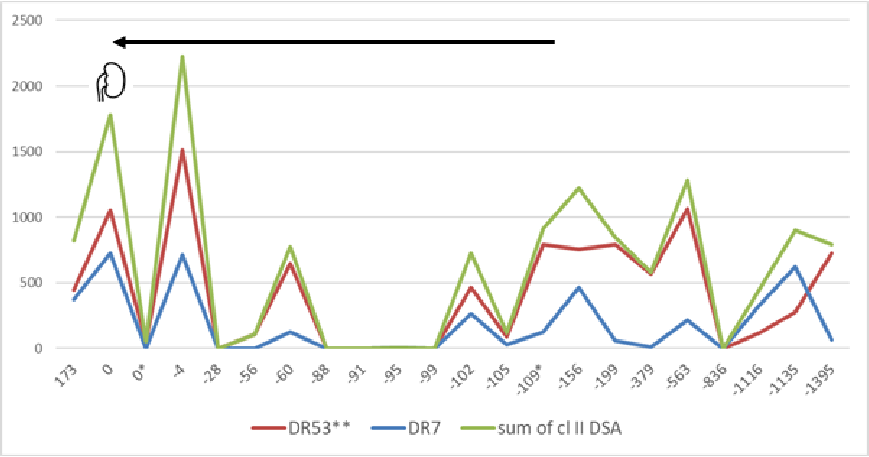

Patient 14

Previous transplantation DSA

HLA Class I

HLA Class II

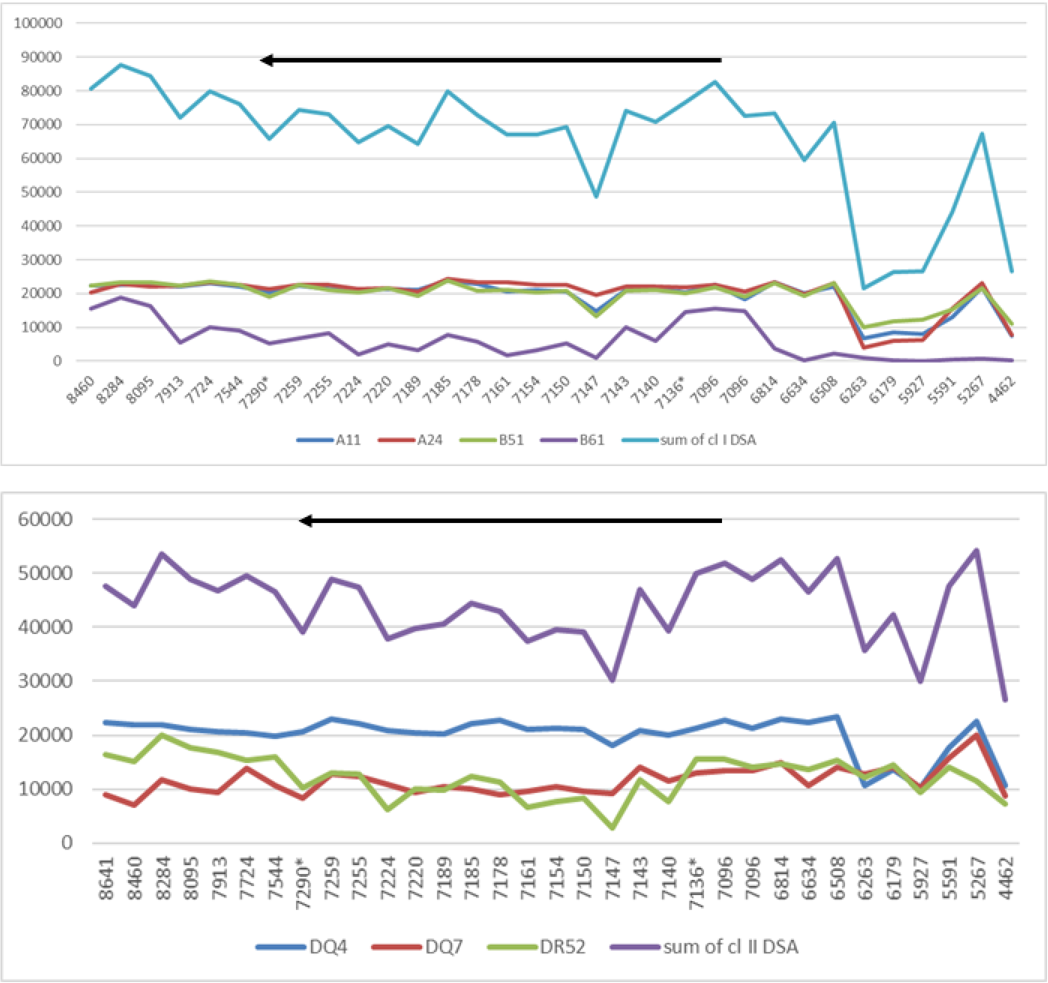

Patient 15 not transplanted
