# Supplementary material for: Immunoadsorption-Based HLA Desensitization in Patients Awaiting Deceased Donor Kidney Transplantation: An Interventional, Non-Randomised, Single Cohort Study
Source: Transpl Int. 2023 Aug 23;36:11212. doi: 10.3389/ti.2023.11212 (PMC10481532; doi:10.3389/ti.2023.11212)
Supplement: Supplementary file 2 [file DataSheet1.docx]

**Immunoadsorption-based HLA desensitization in patients awaiting deceased donor kidney transplantation: an interventional, non-randomised, single cohort study**

Côme BUREAU, Cédric RAFAT, Jean Luc TAUPIN, Stéphanie MALARD, Laurent MESNARD, Hélène FRANCOIS, Camille PETIT-HOANG, Nacera OUALI, Alexandre HERTIG, Matthieu JAMME, David BUOB, Eric RONDEAU, Pierre GALICHON, Yosu LUQUE

**Supplemental Method 1:** Desensitization protocol

**Supplemental Method 2:** Immunoadsorption therapy

**Supplemental Method 3:** Induction and maintenance immunosuppressive therapy for KT

**Supplemental Method 4:** Anti HLA antibodies and CDC crossmatch assessment

**Supplemental Table 1:** Demographics and nephrological characteristics of transplanted patients (individually)

**Supplemental Table 2:** Banff score on the 3rd month protocol biopsy

**Supplemental Table 3:** Post-kidney transplantation infectious complications

**Supplemental Figure 1:** Anti-HLA antibodies trajectories of the patients receiving IADS based desensitization protocol. Arrow start corresponds to IADS protocol start. Kidney symbol corresponds to KT. Flash symbol correspond to ABMR episode.

**Supplemental Method 1:** Desensitization protocol

The desensitization protocol provided for: i) a pharmacological immunosuppressive regimen which included steroid therapy (prednisone, 20 mg/day), tacrolimus 0.2 mg/kg once daily (Advagraf^®^, Astellas, Tokyo, Japan) and mycophenolate mofetil 1 g twice daily (CellCept^®^, Roche, Basel, Switzerland), ii) IADS therapy consisted of a sequence including an induction phase of 5 sessions per week for 2 weeks followed by 3 sessions per week for 2 weeks and a maintenance phase of 3 sessions per week, 1 week per month, until KT was performed. Intravenous immunoglobulins (IVIg) (Privigen^®^, CSL Behring, PA, USA; 1-2 g/kg) were given at immunomodulatory doses, at the end of each week of IADS therapy. The target tacrolimus trough concentration was 6 to 8 ng/mL.

**Supplemental Method 2:** Immunoadsorption therapy

Briefly, IADS sessions consisted of a plasma separation phase achieved via filtration (Art Universal^®^, Fresenius, Bad Homburg, Germany) immediately followed by IADS managed through 2 twin semi-specific staphylococcal protein A columns (Globaffin^®^, Fresenius, Bad Homburg, Germany) using a specific device (ADAsorb^®^, Fresenius, Bad Homburg, Germany).

During each session of IADS, an average of 1.5 plasma volumes (2000–4000 ml) was exchanged.

Anticoagulation of the extracorporeal circuit (EC) was provided through citrate-dextrose solution (ACD) administration aiming for EC ionized calcium concentration of between 0.2 and 0.4 mmol/L. To achieve an adequate chelation of EC calcium, citrate was infused at the ratio of 1 ml/min of ACD for 24 ml/min of blood flow. Calcium (CaCl2) restitution was given through the venous return line at the rate of 1g/ hour.

Whenever possible IADS were performed in tandem with hemodialysis as previously described^10^: using the same Y-shaped needle, the other arterial branch was used to connect the patient to the hemodialysis generator.

We only used post-operative IADS sessions in situations of documented antibody-mediated rejection and not systematically according to DSA rebound.

**Supplemental Method 3:** Induction and maintenance immunosuppressive therapy for KT

Tacrolimus was given in a fixed dose prior to transplantation. All patients received induction therapy at KT consisting of rabbit antithymocyte globulin (Thymoglobulin^®^, Genzyme, Lyon, France) at 1.5 mg/kg for 4 days. Mycofenolate mofetil was continued at the same dose (1 g twice daily). Tacrolimus doses were adjusted to maintain a target trough blood level of 8 to 10 ng/mL for the first 3 months and 6 to 8 ng/mL after 3 months, respectively. A single dose of 500 mg methylprednisolone was administered intravenously on the day of the KT followed by prednisone 20 mg the next day and during the first month, then tapered to 10 mg per day at month 3.

**Supplemental Method 4:** Anti HLA antibodies and CDC crossmatch assessment

Anti HLA antibodies were detected with Luminex^®^ single antigen beads before IADS and then repeatedly monitored every week during IADS-based desensitization in the HLA immunology laboratory, Hôpital St Louis, Paris, France. Donor specific antibody (DSA) detection was performed on the day of KT and at 3 months posttransplant. Patients were cleared for KT following a negative T cell complement-dependent cytotoxicity (CDC) crossmatch when a compatible donor was located. Calculated panel reactive antibody (cPRA) was calculated with the unacceptable antigens (MFI > 2000) using the ETRL virtual PRA calculator. Before IADS protocol, PRA was calculated on neat and diluted sera (ratio 1:10, in negative serum control). PRA (before IA protocol) was also performed on a panel of 30 T lymphocytes (GenTrak®, Liberty, North Carolina, US) according to the manufacturer’s instructions.

*Complement-dependent cytotoxicity crossmatch*: Immunomagnetic beads (Easyse^TM^, Stemcell, Cambridge, UK) were used to separate T and B cells according to the manufacturer’s instructions. 1 µL of donor T and B cells were incubated with 1 µL and 2 µL of recipient sera for 30 min at 22°C, with and without DTT. A 3 µL rabbit complement and staining solution was added and incubated for 60 min. T and B cell CDC crossmatches were considered positive when the observed cell death exceeded 20% above background. Crossmatches by flow cytometry are not performed in deceased donors in France.

*Single Antigen Bead Assays*: Sera were tested using LABScreen^®^ beads from One Lambda, Canoga Park, USA. LABScreen^®^ assays were performed according to the manufacturer’s protocol. Briefly, blood was rapidly centrifuged after sampling, aliquoted, and immediately frozen at -30°C. Before SAFB testing, a 0.1-M solution of disodium EDTA (Sigma-Aldrich^®^, St Louis, USA) at pH=7.4 was diluted 1:10 in the sera and incubated for 10 min to avoid prozone effect. A preliminary adsorption was then performed with microparticles treated with blocking solution to reduce the high background caused by nonspecific binding of materials in human sera to the latex beads used in flow antibody detection assays. 20 µL of test serum was pre-added to 5 µL each of LS1A04 or LS2A01 SA beads, incubated in the dark for 30 min at room temperature, and then washed with wash buffer. 100 µL of goat anti-human Ig G secondary antibody conjugated with R-Phycoerythrin (PE) used at a 1:100 dilution was added to the beads, incubated for 30 min in the dark at room temperature, then washed and read on the LABScan^®^ 100 flow cytometer (One Lambda).

**Supplemental Table 1:** Demographics and nephrological characteristics of transplanted patients (individually)

|  | 1 | 2 | 3 | 4 | 5 | 6 | 7 | 8 | 9 | 10 | 11 | 12 | 13 | 14 |
| --- | --- | --- | --- | --- | --- | --- | --- | --- | --- | --- | --- | --- | --- | --- |
| Sex | F | F | F | F | F | F | F | F | M | M | M | F | F | F |
| Age, years | 52 | 52 | 40 | 52 | 40 | 45 | 36 | 40 | 56 | 60 | 62 | 67 | 53 | 46 |
| Previous kidney transplantations, n | 0 | 2 | 0 | 0 | 0 | 1 | 1 | 1 | 1 | 2 | 1 | 2 | 0 | 1 |
| Kidney disease | Lupus | FSGS | MN | Unknown | IgA N | IgA N | Uropathy | GP | HN | Unknown | HN | Unknown | ADPKD | FSGS |
| Number of pregnancies for women, n | 4 | 5 | 3 | 9 | 3 | 2 | 0 | 1 | NA | NA | NA | 2 | 6 | 5 |
| cPRA, % | 99.9 | 99.4 | 59.5 | 80.8 | 99.2 | 98.8 | 97.6 | 99.1 | 99.9 | 97.6 | 99.7 | 90.8 | 98.2 | 80.6 |
| Duration of renal-replacement therapy, years | 7 | 7 | 4.5 | 6 | 11 | 5 | 5 | 7.5 | 6 | 12 | 6 | 15 | 6 | 10 |
| Time on waiting list, years | 6.5 | 6 | 4 | 5 | 11 | 4 | 5 | 7 | 6 | 12 | 5 | 14 | 5.5 | 9.5 |
| Time from IADS start to transplantation, days | 169 | 132 | 68 | 137 | 7 | 408 | 30 | 142 | 16 | 31 | 236 | 46 | 109 | 110 |
| Donor characteristics |  | | | | | | | | | | | | | |
| Age, years | 68 | 44 | 23 | 70 | 64 | 43 | 37 | 72 | 66 | 79 | 70 | 82 | 37 | 34 |
| Hypertension history | No | No | No | Yes | No | No | No | No | No | No | Yes | Yes | No | No |
| Diabetes mellitus history | Yes | No | No | Yes | No | No | No | No | No | No | No | No | No | No |
| Serum creatinine, µmol/L | 121 | 68 | NA | 85 | NA | 69 | 136 | 53 | 72 | 69 | 64 | 80 | 48 | 31 |
| Proteinuria, g/24h | 0 | 0.01 | NA | 1 | 0 | 0.32 | 0 | 0 | 0 | 0 | 0 | 1 | 0 | 0 |
| Cause of death | CVS | CVS | DCD | CVS | CVS | DCD | DCD | CVS | CVS | CVS | CVS | CVS | DCD | CVS |

**Supplemental Table 1:** Demographic and nephrological characteristics of transplanted patients and donors (individually)

Abbreviations: ADPKD, autosomal dominant polycystic kidney disease; cPRA, calculated panel reactive antibodies; FSGS, focal segmental glomerulosclerosis; GP, Goodpasture disease; HN, hypertensive nephrosclerosis; IADS, immunoadsorption; IgA N, IgA nephropathy; CVS, death due to cerebrovascular cause / stroke; DCD, donor after circulatory death

**Supplemental Table 2 :** Banff score on the 3rd month protocol biopsy

| **Patient** | **Banff score (3-month post-transplant protocol biopsy)** | **C4d** |
| --- | --- | --- |
| **1** | NA | NA |
| **2** | g0i0t0v0cpt0mm0ci0ct0cv0ah1 | 3 |
| **3** | NA | NA |
| **4** | g1i0t0v0cpt1mm0ci0ct0cv0ah0 | 0 |
| **5** | g2i0t0v0cpt0mm1ci1ct1cvNAah0 | 0 |
| **6** | g1i0t0v0cpt0mm0ci0ct0cv0ah1 | 0 |
| **7** | NA | NA |
| **8** | g0i0t0v0cpt0mm0ci1ct1cv2ah1 | 0 |
| **9** | g0i0t0v0cpt0mm0ci0ct0cv0ah0 | 2 |
| **10** | g0i0t0v0cpt0mm0ci1ct1cv2ah2 | 0 |
| **11** | g1i0t0v0cpt0mm0ci0ct0cv1ah0 | 3 |
| **12** | NA | NA |
| **13** | NA | NA |
| **14** | NA | NA |

Abbreviations: g, glomerulitis; i, interstitial inflammation; t, tubulitis; v, intimal arteritis; cpt, peritubular capillaritis; cg, transplant glomerulopathy; mm, mesangial matrix increase; ci, interstitial fibrosis; ct, tubular atrophy; cv, arterial fibrous intimal thickening; ah, hyaline arteriolar thickening. NA: non-available.

C4d staining is performed by immunohistochemistry on paraffin embedded sections.

**Supplemental Table 3***:* Post-kidney transplantation infectious complications

| **Patient** | **Number of IADS** | **Prior IS** | **Type of infection** | **Severity criteria on presentation** | **Delay between KT -infection** | **Pathogen documentation** | **Site of infection** | **Coinfection** | | **Outcome** | | **Anti-infectious therapy** | | **Immunosuppression modification** |
| --- | --- | --- | --- | --- | --- | --- | --- | --- | --- | --- | --- | --- | --- | --- |
| **1** | 32 | IS | Invasive aspergillosis | Multiorgan failure | M1 | *Aspergillus Flavus* | Respiratory tract  Bloodstream | 1) Undocumented bacterial coinfection  2) CMV reactivation | | Death | | Caspofungin | | PE and steroids for ABMR |
| **2** | 65 | KT |  | | | | | | | | | | | |
| **3** | 26 |  | Abscess | Transplantectomy  SSI | M1 | *Enterococcus Faecium* | Transplantectomy  SSI | None | |  | | Surgery  Vancomycin | | None |
| **4** | 33 |  |  | | | | | | | | | | | |
| **5** | 5 |  | BSI | BSI | M5 | *Pseudomonas aeruginosa* | Urinary tract | None | | 127 µmol/l Serum creatinine  Stable KGF | | Meropenem | | Stop MMF and Prednisone |
|  |  |  | Disseminated  Cryptococcosis | Stupor | M8 | *Cryptococcus neoformans* | Cutaneous  CNS  Respiratory tract |  |  | Stable KGF  No neurological sequelae | | 1)Ampho B + 5-FC  2)Fluconazole | |  |
|  |  |  | Pyelonephritis | Abscess  BSI | Y4 | *Klebsiella pneumoniae* | Urinary tract |  |  | Stable KGF | | 1)Cefotaxime+Ofloxacin  2)Ofloxacin | |  |
| **6** | 39 | KT |  |  |  |  |  |  |  |  |  |  |  |  |
| **7** | 13 | KT |  |  |  |  |  |  |  |  |  |  |  |  |
| **8** | 22 | KT/IS | Pyelonephritis | None | Y1 | Unknown | Urinary tract | RSV upper airway infection | Stable KGF | | | Meropenem | | Eculizumab |
| **9** | 13 | KT | Diarrhea | VP support  KDIGO 3 AKI | M2 | *Norovirus* | Colon | CMV reactivation | 30 % degradation of KGF | | | Immunosuppression modulation | | None |
|  |  |  | Pyelonephritis | VP support  BSI  KDIGO 2 AKI | Y2 | *Escherichia coli* | Urinary tract |  | Stable KGF | | | Cefotaxime-Metronidazole -Amikacin | |  |
| **10** | 15 | KT | Transport fluid contamination related mycotic aneurysm | Mycotic aneurysm  Transplantectomy | D0 🡪 Y1 | *Candida krusei*,  *Candida albicans*  *Staphylococcus epidermidis* | Graft artery | None | Mycotic aneurysm  Degradation of KGF | | | 1)Caspofungin  2)Graft artery angioplasty  3)Transplantectomy | | None |
|  |  |  | Bacterial colitis | KDIGO 3 AKI | M3 and Y1 | *Norovirus*  *Campylobacter jejuni* | Colon |  | 25% | | | Urine deviation  Ciprofloxacin  decreased immunosuppression | | None |
| **11** | 24 | KT |  |  |  |  |  |  |  |  |  |  |  |  |
| **12** | 16 | KT | Invasive aspergillosis | MV  KDIGO 3 AKI | Y1 | *Aspergillus fumigatus*  *Pseudomonas Aeruginosa*  *ESBL Klebsiella pneumoniae* | Respiratory tract  Urinary tract | None | Death | | 1)Meropenem- Rovamycine- Amikacin  Ampho B 2)Voriconazole | | None | |
| 13 | 21 |  | Septicemia | BSI  KDIGO 2 AKI | M6 | *Enterobacter cloacae* | Urinary tract | None | None | | 1)Tazocillin  2)Imipenem  Urine deviation | | None | |
| 14 | 25 |  | Influenza | High flow oxygenation | M4 | H3N1 influenzae | Respiratory tract | CMV reactivation | Recurrent FSGS  Graft loss | | 1)Ciprofloxacin- 2)Meropeneme – 3)Linezolid Oseltamivir | | Steroid therapy  Cyclosporine  Ofafumumab  PE  Neutropenia | |

Numbered list denotes sequential therapy

Abbreviations: AKI, acute kidney injury; ABMR, antibody mediated rejection; BSI, bloodstream infection; CMV, cytomegalovirus; CNS, central nervous system; FSGS, focal segmental glomerulosclerosis; 5 FC, flucytosine; IADS, immunoadsorption; IS immunosuppression; KDIGO, Kidney Disease: Improving Global Outcomes; KGF, kidney graft; KT, kidney transplantation; MMF, mycophenolate mofetil; PE, plasma exchange; SSI, surgical site infection; RSV, respiratory syncytial virus
